# Boosted Enzyme Activity via Encapsulation within Metal–Organic Frameworks with Pores Matching Enzyme Size and Shape

**DOI:** 10.1002/advs.202309243

**Published:** 2024-04-04

**Authors:** Ying Liu, Ziman Chen, Zheng Wang, Yongqin Lv

**Affiliations:** ^1^ State Key Laboratory of Organic‐Inorganic Composites National Energy Research and Development Center for Biorefinery International Joint Bioenergy Laboratory of Ministry of Education Beijing Key Laboratory of Bioprocess College of Life Science and Technology Beijing University of Chemical Technology Beijing 100029 China

**Keywords:** boosted enzyme activity, enzyme immobilization, metal–organic frameworks, size and shape complementarity

## Abstract

A novel and versatile approach called “physical imprinting” is introduced to modulate enzyme conformation using mesoporous materials, addressing challenges in achieving improved enzyme activity and stability. Metal–organic frameworks with tailored mesopores, precisely matching enzyme size and shape, are synthesized. Remarkably, enzymes encapsulated within these customized mesopores exhibit over 1670% relative activity compared to free enzymes, maintaining outstanding efficiency even under harsh conditions such as heat, exposure to organic solvents, wide‐ranging pH extremes from acidic to alkaline, and exposure to a digestion cocktail. After 18 consecutive cycles of use, the immobilized enzymes retain 80% of their initial activity. Additionally, the encapsulated enzymes exhibit a substantial increase in catalytic efficiency, with a 14.1‐fold enhancement in *k*_cat_/*K*_M_ compared to native enzymes. This enhancement is among the highest reported for immobilized enzymes. The improved enzyme activity and stability are corroborated by solid‐state UV–vis, electron paramagnetic resonance, Fourier‐transform infrared spectroscopy, and solid‐state NMR spectroscopy. The findings not only offer valuable insights into the crucial role of size and shape complementarity within confined microenvironments but also establish a new pathway for developing solid carriers capable of enhancing enzyme activity and stability.

## Graphical abstract

Using a physical imprinting technique, mesoporous metal–organic framework (MOF) materials imparting exquisite enzyme‐specificity are successfully synthesized. Physical imprinting produced MOF architectures containing intricate pores precisely complementing the size and shape of target enzymes. Enzymes encapsulated within these customized mesopores demonstrate preserved activity and stability, retaining native‐like properties over extended operation.

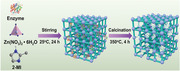

## 1 Introduction

Enzymes represent one of nature's most effective classes of biocatalysts, demonstrating remarkable regio‐ and chemoselectivity to delicately regulate living processes under mild conditions. Over evolutionary time, a dazzling array of enzymes has emerged to perform virtually any transformation necessary to sustain life.^[^ ^1^ ^]^ While enzymes demonstrate profound effectiveness within cellular environments, their function is also tethered to fragility. Most operate only within the narrow protected zone of the cell, rapidly losing tertiary structure and catalytic abilities outside of this milieu. The intricate globular formation of these molecules, characterized by high dynamism and sensitivity to temperature, enables them to carry out biochemistry with remarkable precision. However, this very dynamic nature also makes them susceptible when exposed to environments outside their native habitats. For pragmatic applications, therefore, stabilization approaches are required to preserve the delicate architectures and activities of these workhorse proteins, harnessing their potential while shielding their sensitivities from destabilizing influences that could otherwise compromise function.^[^ ^2^ ^]^

While immobilization often enhances operational stability through facile recovery and removal from hostile external influences, the technique can concomitantly diminish catalytic prowess from confining surface interactions. Yet emerging studies reveal how rare immobilization matrices may instead enhance enzyme activity beyond native levels through judicious regulatory effects.^[^ ^3^ ^]^ Among the most promising supports, metal–organic frameworks (MOFs) and covalent‐organic frameworks (COFs) offer exquisitely tunable chemistries and pore architectures.^[^ ^3^, ^4^ ^]^ While encouraging initial findings demonstrate activity boosts through tailored MOF encapsulation,^[^ ^3^, ^5^ ^]^ knowledge of the conformational ramifications remains limited, especially regarding the impact of shape and size complementarity between rigid scaffold and flexible biocatalyst.

Herein, we introduce a novel “physical imprinting” synthesis of mesoporous zeolitic imidazolate framework‐8 (ZIF‐8) engineered to match model protein templates at the nanoscale. After autonomously imprinting size‐shaped cavities coordinating four model proteins, calcination yields the remaining porous matrix. Remarkably, enzymes reconstituted within these customized mesopores mirrored the native imprints, conferring unprecedented activity and stability enhancements under stressful conditions. Through multi‐spectroscopic interrogation, we systematically unveil how exquisite conformational regulation stems from intimate shape‐complementary confinement. Overall, these results establish a biomimetic design paradigm for optimizing immobilization to safeguard enzymatic domains through nanoscale topographical synergy between carrier and biocatalyst.

## 2 Results and Discussion

### 2.1 Creating Mesopores in ZIFs via Physical Imprinting Strategy

**Scheme 1:**
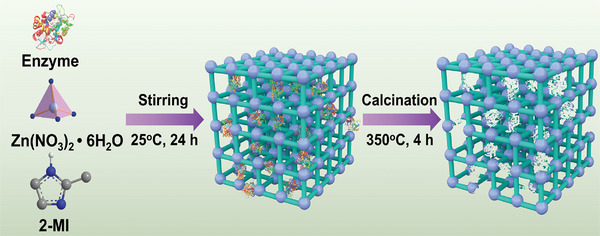
Schematic illustration depicting the process for the preparation of HZIF‐8_[Enzyme]_ through a “physical imprinting” technique.

As illustrated in **Scheme** **1**, the enzyme‐embedded ZIF (enzyme‐ZIF‐8 composite) was initially synthesized using a “one‐pot” co‐precipitation approach, wherein Zn(II) ions coordinated with 2‐methylimidazole in the presence of enzymes. During this process, the enzyme actively participates in the self‐assembly via coordination with Zn(II) ions. Following the formation of the enzyme‐ZIF‐8 composite, the enzyme template was eliminated through calcination, resulting in tailored mesopores that precisely match the size and shape of the enzyme.

**Figure 1:**
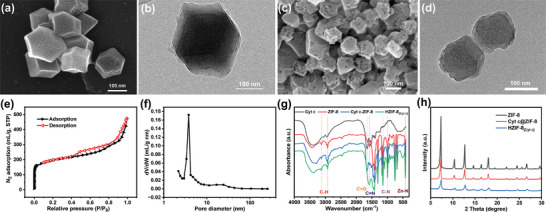
a) SEM image of Cyt c@ZIF‐8 composite; b) TEM image of Cyt c@ZIF‐8 composite; c) SEM image of HZIF‐8_[Cyt c]_; d) TEM image of HZIF‐8_[Cyt c]_; e) N_2_ adsorption/desorption isotherms of HZIF‐8_[Cyt c]_; f) Pore size distribution of HZIF‐8_[Cyt c]_; g) FTIR spectra of Cyt c, ZIF‐8, Cyt c@ZIF‐8 composite, and HZIF‐8_[Cyt c]_; h) PXRD of ZIF‐8, Cyt c@ZIF‐8 composite, and HZIF‐8_[Cyt c]_.

To demonstrate our concept, we initially used cytochrome c (Cyt c) as the template to create enzyme‐adapted mesoporosity in ZIF‐8. Thermogravimetric analysis identified 350 °C as the optimal calcination temperature to remove Cyt c while preserving ZIF‐8 integrity (Figure S1a, Supporting Information). Scanning electron microscopy (SEM) and transmission electron microscopy (TEM) images of the Cyt c‐ZIF‐8 composite (**Figure** **1**a,b) displayed a rhombic dodecahedron morphology, resembling the ZIF‐8 prepared using the conventional solvothermal method.^[^ ^6^ ^]^ Following the calcination step, the Cyt c template was removed, resulting in HZIF‐8_[Cytc]_ with an identical morphology and hierarchical micro‐ and mesoporous structure (Figure 1c,d). High‐resolution TEM of HZIF‐8 visualized the imprinted mesopores as low‐scattering light spots. Nitrogen physisorption experiments on HZIF‐8_[Cytc]_ generated a type‐I and type‐IV isotherm (Figure 1e) denoting coexistent micro‐ and mesoporosity, distinguished from the conventional microporous ZIF‐8.^[^ ^4k^ ^]^ The mesopores facilitated efficient enzyme encapsulation through hydrophobic interactions between the MOF cages’ hydrophobic walls and enzyme molecules, while the micropores facilitated rapid mass transport of substrates and products. Pore size analysis reported an average 7.90 nm mesopore diameter and 0.48 cm^3^ g^−1^ mesopore volume (Figure 1f; Table S1, Supporting Information), confirming successful mesopore incorporation.

The Fourier transform infrared spectroscopy (FTIR) spectrum of the Cyt c‐ZIF‐8 composite (Figure 1g) revealed prominent absorption bands associated with the structural properties of the material. Peaks at 3134 and 2928 cm^−1^ correlated with aromatic and aliphatic C─H stretching vibrations emanating from the imidazole moieties. The characteristic peak at 1650 cm^−1^ was attributed to the carboxyl acid groups of Cyt c. The 1579 cm^−1^ peak indicated the stretching of the C═N bonds, while those at 995 and 1137 cm^−1^ pointed to C─N bond stretches. The 421 cm^−1^ feature implied Zn─N stretching vibrations. These characteristic signatures corroborated the successful synthesis of ZIF‐8. Additionally, the prominent 1650 cm^−1^ band evidenced the successful incorporation of Cyt c within the ZIF‐8 framework. Following calcination, all characteristic ZIF‐8 peaks remained discernible, with the exception of the 1650 cm^−1^ feature, confirming the efficient removal of the Cyt c templating agent and yielding of HZIF‐8_[Cytc]_. Furthermore, Figure 1h revealed the calcined material retained the high crystallinity and parallel powder X‐ray diffraction pattern of the parent Cyt c@ZIF‐8 and ZIF‐8. This indicated the calcination process did not degrade the excellent crystallinity or topology of the underlying ZIF‐8 scaffold.

We investigated the decomposition mechanism of the Cyt c‐ZIF‐8 composite and conventional ZIF‐8 by analyzing their mass loss behavior and gas evolution properties using online thermogravimetry‐mass spectrometry (TG‐MS). Figure S2 (Supporting Information) in the supporting information presents the gradual heating of the Cyt c‐ZIF‐8 composite to 350 °C, resulting in the significant release of H_2_O, NO, and CO_2_ gases. These gases were attributed to the organic nature of Cyt c. In contrast, the generic ZIF‐8 exhibited no gas release at 350 °C, indicating its thermal stability during the calcination process. To further validate this finding, we examined the high‐resolution TEM image of generic ZIF‐8 after calcination. As depicted in Figure S1b (Supporting Information), no mesopores were observed, highlighting the crucial role of the enzyme template in the formation of mesopores.

To demonstrate the versatility of our novel physical imprinting method, we prepared hierarchically micro‐ and mesoporous HZIF‐8_[HRP]_ and HZIF‐8_[Lipase]_ using similar synthetic procedures, replacing the Cyt c template with horseradish peroxidase (HRP) or lipase. The morphology and microstructure of HRP‐ZIF‐8, HZIF‐8_[HRP]_, lipase‐ZIF‐8, and HZIF‐8_[Lipase]_ were analyzed using various physical and spectroscopic techniques, as depicted in Figures S3 and S4 (Supporting Information). SEM images revealed that HZIF‐8_[HRP]_ and HZIF‐8_[Lipase]_ shared a similar rhombic dodecahedral morphology, and their TEM images demonstrated the formation of mesopores.

The corresponding N_2_ adsorption/desorption isotherms revealed type‐I and type‐IV characteristics, indicating the coexistence of micro‐ and mesopores in HZIF‐8_[HRP]_ and HZIF‐8_[Lipase]_. Larger enzyme templates created proportionally expanded mesoporosity, optimizing matrix accommodation of biomolecular cargo. For example, the HZIF‐8_[HRP]_ synthesized using HRP as the template exhibited a mesopore size of 11.89 nm and a mesopore volume of 0.51 mL g^−1^, while HZIF‐8_[Lipase]_ prepared with the presence of lipase template displayed a mesopore size of 15.04 nm and a mesopore volume of 0.88 mL g^−1^. Their BET surface areas were notably reduced due to the increased number of mesopores. Notably, the average mesopore size of HZIF‐8 exceeded the dimensions of the enzyme template molecules, possibly due to enzyme aggregation during the formation of enzyme‐embedded MOFs. To gain deeper insights into enzyme aggregation, we conducted a comprehensive analysis of pore volume distribution across different pore sizes in HZIF‐8_[enzyme]_, as detailed in Tables S2–S5 (Supporting Information). Notably, Cyt c, lipase, and BSA exhibited similar proportions of pore volume (ranging from 32.8% to 36.7%) created by their respective monomers, except for HPR, which demonstrates a significantly lower proportion of pore volume (20.5%) generated by its monomer. Furthermore, the four enzymes vary in their aggregation states, with HRP revealing a much higher proportion (52.5%) in its higher‐order oligomer configurations. This indicates that HRP exhibits a greater tendency for aggregation during MOF formation when compared to the other enzymes. These tailored mesopores created by monomers precisely match the enzyme size and shape, contributing to the enhancement of enzyme activity and stability. Additionally, the larger pores formed by aggregated enzymes facilitate rapid mass diffusion during the enzyme immobilization process.

The characteristic stretching vibrations of C─H (3131 and 2928 cm^−1^), C═N (1583 cm^−1^), and C─N (991 and 1141 cm^−1^) for imidazole groups, as well as Zn─N (421 cm^−1^) for the coordination between Zn^2+^ and imidazole, were observed in HZIF‐8_[HRP]_ and HZIF‐8_[Lipase]_, confirming the successful formation of zeolitic imidazolate frameworks. The prominent band at 1662 cm^−1^ (C═O) in HRP‐ZIF‐8 and lipase‐ZIF‐8 disappeared after calcination, indicating the effective removal of the enzyme template. The PXRD patterns validated the highly crystalline structures of HZIF‐8_[HRP]_ and HZIF‐8_[Lipase]_, consistent with those of HZIF‐8_[Cytc]_ and conventional microporous ZIF‐8.

### 2.2 Immobilization of Enzymes in HZIF‐8

**Figure 2:**
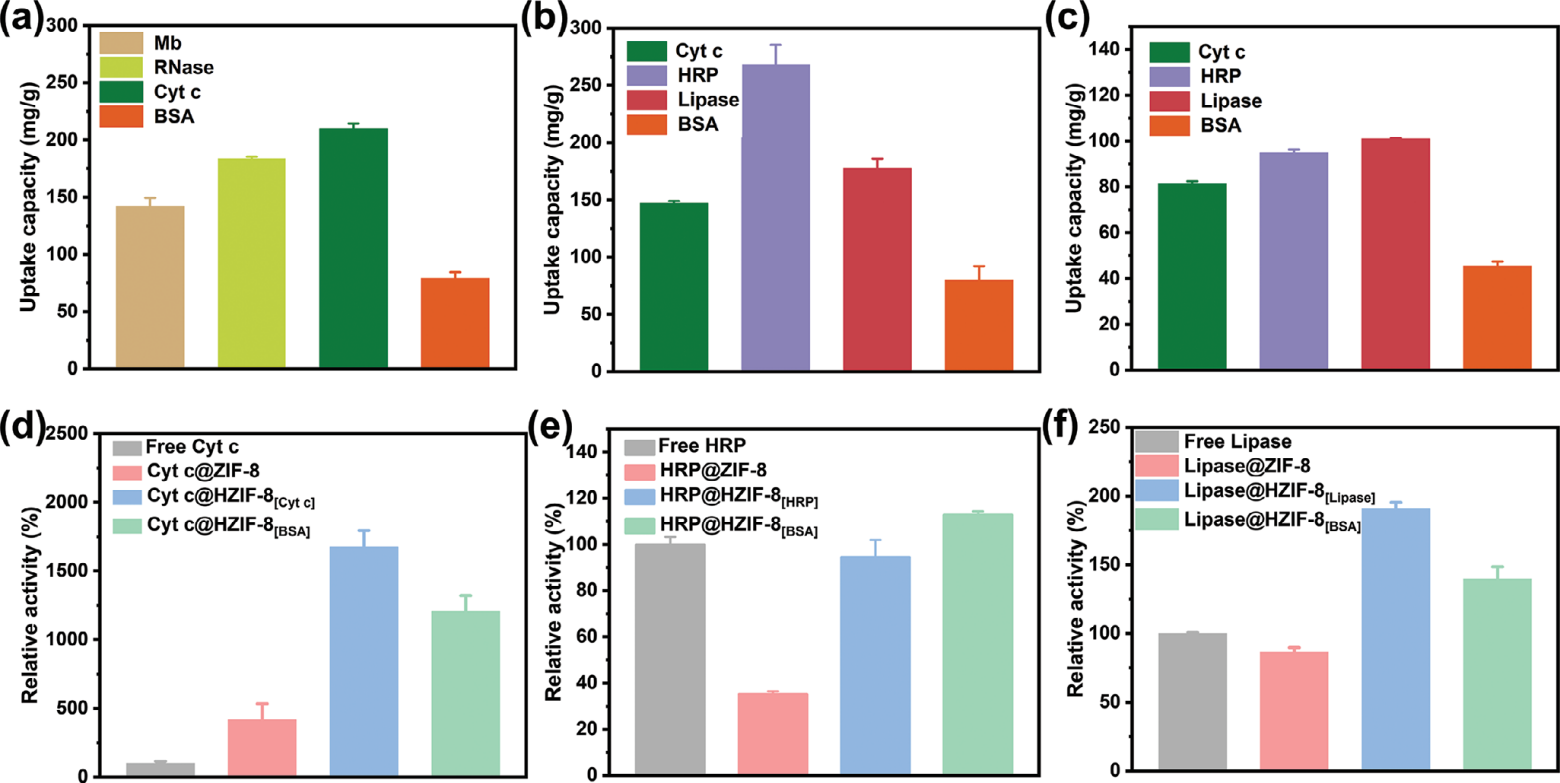
The uptake capacity of a) HZIF‐8_[Cyt c]_, b) HZIF‐8_[HRP]_, and c) HZIF‐8_[Lipase]_ for Mb, RNase, Cyt c, HRP, lipase, and BSA; The relative activity of d) free and immobilized Cyt c, e) free and immobilized HRP, and f) free and immobilized lipase. The relative activity pertains to the enzyme activity in reference to the free enzyme, which has been normalized to 100%.

Our results provide compelling evidence that physical imprinting enables scalable synthesis of MOFs tailored precisely to enzymes. When assessing the uptake capacities of varied proteins within our imprinted ZIF matrices, uptake correlated strongly to size complementarity between mesopores and cargo (**Figure** **2**a–c). Intriguingly, each HZIF‐8 variant exhibited the highest capacity selectively for the templating protein. For instance, HZIF‐8_[Cytc]_ incorporated 209.4 mg g^−1^ Cyt c compared to lower, size‐dependent immobilization of other payloads. Similarly, HZIF‐8_[HRP]_ encapsulated 267.6 mg g^−1^ HRP versus diminished quantities of alternate cargoes. HZIF‐8_[Lipase]_ captured 101.1 mg g^−1^ lipase preferentially. The marked preference reflects energetically favorable nanoconfinement within shape‐complementary interfaces, emphasizing physical imprinting's ability to synthesize solid hosts optimized for designated biomolecular targets. Notably, the larger BSA exhibited diminished uptake across matrices consistent with steric exclusion from nanocavities. For example, the encapsulated capacity of HZIF‐8_[BSA]_ was 135.3 mg g^−1^ for Cyt c, 105.3 mg g^−1^ for HRP, and 72.5 mg g^−1^ for lipase (Figure S5, Supporting Information). Our findings demonstrate that members of the HZIF‐8 family can now be custom‐synthesized as solid carriers for enzymes, featuring specificity directed toward templated protein targets.

Incorporating enzyme “templates” during ZIF synthesis orchestrates the formation of the framework, as the mimetic cavities of the enzyme guide the spatial organization of zinc and imidazole nodes around its 3D structure. The enzyme acts as a scaffold, directing the topological wrapping of nodes to align with its configuration. Upon template removal, the porous architecture retains the imprint of the enzyme, manifesting as matching cavities and pores. This physical imprint establishes size and shape complementarity between resulting mesopores and the templating enzyme. Upon introducing alternate cargo molecules, only payloads possessing a size, shape, and surface topology akin to the original template efficiently occupy the imprinted cavities. This snug fit optimizes attractive van der Waals and hydrophobic interactions at the solid‐fluid interface. Bulkier species are sterically hindered, and those with mismatching surface features lack the same binding affinity. Consequently, energetically favorable binding occurs selectively for the templating enzyme.

High‐resolution SEM images of HZIF‐8 after enzyme encapsulation showed no discernible change in MOF morphology compared to HZIF‐8_[enzyme]_ (Figures S6 and S7, Supporting Information). Moreover, high‐resolution TEM provided corroborating evidence of intact enzyme encapsulation within the mesoporous imprints, rather than superficial adsorption onto external surfaces (Figure S8, Supporting Information). Elemental dispersion mapping derived from energy‐dispersive X‐ray spectroscopy further demonstrated the uniform intracellular distribution of enzymatic cargoes (Figures S9–S11, Supporting Information). Furthermore, confocal laser scanning microscope (CLSM) images indicated that the three enzymes were evenly distributed inside HZIF‐8, further confirming the enzyme's encapsulation (Figures S12–S14, Supporting Information). Gratifyingly, immobilization did not induce distortions to the underlying frameworks’ architectures. These analytical findings validate that the physical imprinting approach effectively synthesizes biomimetic scaffolds with hospitable qualities, capable of intimately accommodating high payloads of enzyme guests in a manner reminiscent of natural cellular environments.

### 2.3 Catalytic Performances of Enzymes Encapsulated in HZIF‐8

Before examining the catalytic performances of immobilized enzymes, we conducted an assessment to determine if the encapsulated enzymes leaked out of HZIF‐8 during the catalytic process. The enzymes immobilized in HZIF‐8 were subjected to incubation in a buffer solution, and at regular intervals, we measured enzyme concentrations in the supernatant. Throughout all incubations, there was no notable presence of enzyme detected in any supernatant sample. This finding indicates that the enzymes were effectively retained within HZIF‐8 and did not undergo leakage over the studied time period.

Evaluating the catalytic prowess of enzymes encapsulated within our customized HZIF matrices, we monitored absorbance changes for model enzymatic reactions using free, de novo immobilized, and physically imprinted variants (Figure 2d–f). The specific activity of Cyt c was evaluated through its reduction of 2,2′‐azino‐bis(3‐ethylbenzothiazoline‐6‐sulphonic acid) (ABTS), as monitored by increased absorbance at 415 nm due to ABTS radical formation. Similarly, HRP activity was determined via its peroxidation of guaiacol, quantified by absorbance measurements of tetra‐guaiacol production at 436 nm. Lipase activity was assessed through hydrolysis of 4‐nitrophenyl laurate, with lipase‐catalyzed production of *p*‐nitrophenol quantitated at 410 nm. Strikingly, Cyt c within HZIF‐8_[Cytc]_ exhibited a 1674% relative activity compared to 100% of free enzyme and 417% of the de novo constructed Cyt c‐ZIF‐8 composite. To the best of our knowledge, this value represents the highest enhancement of enzyme activity when compared with previously reported for immobilized Cyt c in porous organic frameworks (refer to Table S6, Supporting Information).^[^ ^3^, ^5^ ^]^ Likewise, the encapsulated lipase in HZIF‐8_[Lipase]_ demonstrated a relative activity of 191% compared to 100% for the native enzyme and 86.4% for the lipase@ZIF‐8 composite. The relative activity of encapsulated HRP in HZIF‐8_[HRP]_, however, was 94.5% compared to 100% observed for free HRP and 35.1% for HRP immobilized in HZIF‐8_[BSA]_. As previously discussed, our analyses revealed that HRP exhibited a greater tendency for aggregation during MOF synthesis when compared to the other three enzymes. The tailored mesopores, engineered through the templating of enzyme monomers, inherently contribute to the enhancement of enzyme activity and stability. This explanation sheds light on the lower activity observed for HRP@HZIF‐8_[HRP]_.

Delving deeper, we assessed kinetic parameters *k*_cat_ and *K*_M_ for both free enzymes and the immobilized enzymes in HZIF‐8 (Table S6 and Figures S15–S17, Supporting Information). Remarkably, the encapsulated Cyt c in HZIF‐8_[Cytc]_ exhibited a significant reduction in the *K*_M_ value (from 183.1 to 84.4 µm), correlating to a 2.2‐fold enhancement in substrate affinity compared to the free enzyme, likely through prereacted substrates concentrated within intimate mesopores.^[^ ^3^, ^7^ ^]^ Meanwhile, the increase in *k*_cat_ (from 0.4 to 2.6 s^−1^) of immobilized Cyt c suggested its enhanced enzyme activity, consistent with the activity data in Figure 2d. Collectively, this conferred a stunning 13.3‐fold amplified catalytic efficiency (*k*_cat_/*K*_M_) versus free Cyt c. Similarly, both lipase and HRP benefited from lowered *K*_M_ and elevated *k*_cat_ within their customized environments, surpassing native kinetic characteristics. Lipase@HZIF‐8_[Lipase]_ achieved the highest reported 14.1‐fold efficiency surge versus free lipase, compared to alternative frameworks (refer to Table S6, supporting information).^[^ ^3^, ^5^, ^8^ ^]^ These results compellingly demonstrate how physical imprinting synthesizes biomimetic scaffolds exquisitely matched to redirect enzymatic dynamics, profoundly activating intrinsic catalysis through congruent nanoscale synergy between immobilization matrix and biocatalyst cargo. The customized mesopores seamlessly navigate substrates while stabilizing active conformations, illuminating new avenues for bioengineering optimized biocatalytic systems.

### 2.4 Increased Stability and Reusability

**Figure 3:**
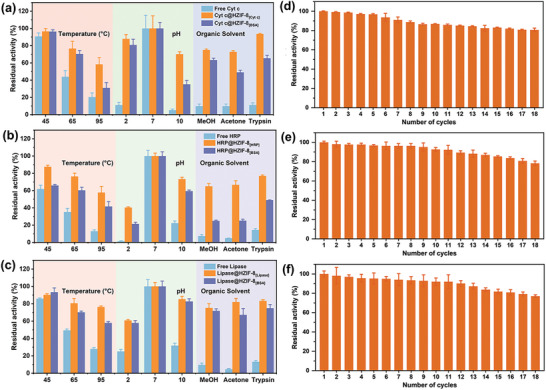
The thermal and chemical stabilities of a) free Cyt c, Cyt c@HZIF‐8_[Cyt c]_, and Cyt c@HZIF‐8_[BSA]_; b) free HPR, HRP@HZIF‐8_[HRP]_, and HRP@HZIF‐8_[BSA]_; c) free lipase, lipase@HZIF‐8_[Lipase]_, and lipase@HZIF‐8_[BSA]_ against elevated temperatures, acidic or alkaline conditions, and organic solvents. The operational stability of Cyt c@HZIF‐8_[Cyt c]_, HRP@HZIF‐8_[HRP]_, and lipase@HZIF‐8_[Lipase]_ after undergoing 18 cycles of repeated use. The relative activity pertains to the enzyme activity in reference to the free enzyme, which has been normalized to 100%.

Assessing enzyme durability under hostile conditions illuminates the protective benefits of physical imprinting methodology (**Figure** **3**). Unsurprisingly, solutions of free Cyt c exhibited marked inactivation at elevated temperatures or across pH extremities that denature native structures. However, within optimally matched mesopores, Cyt c@HZIF‐8_[Cytc]_ retained activity 2.3‐fold higher at 65 °C and 1.9‐fold at 95 °C compared with free Cyt c. Strikingly, even under strong acidity or alkalinity, imprinted Cyt c withstood conditions obliterating free counterparts. For example, Cyt c@HZIF‐8_[Cytc]_ retained 87.9% and 70% of its initial activity at pH 2 and 10, respectively, whereas the free enzymes retained only 11.2% and 5.2% of its initial activity. Likewise, organic solvents decomposing free enzyme showed diminished effects on the stabilized immobilized variant, maintaining 75% and 72.8% of its activity versus near‐complete deactivation. Imprinted Cyt c also resisted proteinase, retaining 93.6% of its original activity in comparison to only 11.2% activity for the native counterpart. Similar protective enhancements benefited HRP and lipase within their respective tailored matrices (Figure 3b,c). These findings powerfully confirm customized confinement mimicking protective cellular machinery. Further, high reusability demonstrated the practicality of physical imprinting for industrial applications (Figure 3d–f). Even after exhaustive 18 consecutive reaction cycles, imprinted biocatalysts retained outstanding 77–80% residual activities. By sensitively optimizing the onboarding environment, our approach synthesizes structurally reinforced, hyper‐resilient biocatalytic nanosystems retaining full prowess across demanding operational conditions, a transformational methodology for developing sustainable biomanufacturing processes.

### 2.5 Importance of Size and Shape Complementary of Mesopores in HZIF‐8

To further probe the significance of architectural matching between matrix and enzyme, we synthesized HZIF‐8_[BSA]_ imprinted with the relatively large 66.4 kDa protein, bovine serum albumin (BSA) (Figure S18, Supporting Information). As expected, resultant 20.15 nm mesopores better accommodated BSA but exhibited reduced surface area versus smaller templated variants (Table S1, Supporting Information). This BSA‐adapted matrix demonstrated a size‐selection effect. Immobilizing smaller Cyt c, HRP, and lipase revealed occupancy declined 1.9‐, 3‐, and 1.8‐fold from their optimized frameworks (Figure 2a–c). The enlarged mesopores imposed a mismatch disrupting shape‐shape interactions. Nonetheless, the enzymes immobilized in HZIF‐8_[BSA]_ still displayed improved enzyme activity compared to their free counterparts in solution. Specifically, Cyt c@HZIF‐8_[BSA]_, HRP@HZIF‐8_[BSA]_, and lipase@HZIF‐8_[BSA]_ demonstrated 1.2‐, 1.1‐, and 1.4‐fold increased activity, respectively. However, the degree of increased activity observed in HZIF‐8_[BSA]_ was comparatively lower than that observed in the custom‐made HZIF‐8. Further kinetic investigation revealed a persistence of tuning effects, although to a more modest extent without the benefit of intimate size‐matching between scaffold and enzyme guest. The stability studies resembled those of the optimized imprints, with the BSA formulations demonstrating improved resistance to heat, varied pH environments, organic solvent conditions, and tryptic digestion when compared to their free enzyme counterparts (Figure 3). However, iterative reusability tests revealed a subtle divergence. While HZIF‐8_[BSA]_ sustained enhanced robustness, performance declined marginally faster than customized cages. Together, these data compellingly demonstrate that size/shape complementarity fine‐tunes activity/stability synergies through precisely interdigitating host‐guest interfaces.

### 2.6 Mechanism of Enhanced Enzyme Activity

**Figure 4:**
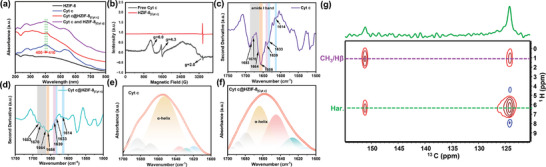
a) Normalized diffuse reflective UV−Vis spectra of HZIF‐8, free Cyt c, Cyt c@HZIF‐8_[Cyt c]_, and the physical mixture of Cyt c and HZIF‐8_[Cyt c]_. b) Normalized EPR spectra of free Cyt c and Cyt c@HZIF‐8_[Cyt c]_ collected at 90 K. Second derivative of the ATR‐FITR spectra of c) Cyt c and d) Cyt c@HZIF‐8_[Cyt c]_. Peak fitting of the Amide I band in the ATR‐FITR spectra of e) Cyt c and f) Cyt c@HZIF‐8_[Cyt c]_. Shadow color scheme: β‐turn, gray; α‐helix, orange; random coil, pink; β‐sheet, blue; Intermolecular β‐sheet, green. g) 2D ^13^C‐^1^H HETCOR NMR spectra of Cyt c@HZIF‐8_[Cyt c]_.

To elucidate the mechanism of enhanced activity of enzymes encapsulated in HZIF‐8, we conducted solid‐state UV–vis spectroscopy to examine the heme active center of Cyt c. As depicted in **Figure** **4**a, the Soret band of Cyt c in HZIF‐8@Cyt c exhibited a redshift from 400 nm to 410 nm, whereas this characteristic peak remained unchanged when HZIF‐8 and Cyt c were physically mixed together. This intriguing finding suggests a significant alteration in the microenvironment surrounding the active center of Cyt c following its encapsulation within HZIF‐8.^19 [^ ^3k^ ^]^

Electron paramagnetic resonance (EPR) spectroscopy was utilized to assess the perturbations of the heme active center in Cyt c upon confinement. The native enzyme displayed a prominent peak at g = 6 (115 mT) and 4.3 (156 mT), representing high spin state with axial symmetry and rhombic symmetry, respectively. However, the immobilized Cyt c within HZIF‐8_[Cyt c]_ demonstrated a prominent g = 2.0 signal diverging from solution counterparts (Figure S6b, Supporting Information). This phenomenon can be ascribed to the interplay between Cyt c and ZIF‐8, as well as the generation of protein‐derived radicals originating from amino acid residues when constricted within a constrained spatial milieu.^[^ ^3^, ^9^ ^]^

Turning to structural analyses, FTIR spectra of free Cyt c feature a diagnostic amide I band ranging from 1600 to 1700 cm^−1^ (Figure 4c), primarily attributed to the stretching vibration of the C═O bonds. This band is highly sensitive to alterations in protein conformation. A more detailed analysis of the secondary structures was conducted by utilizing the second derivative of the amide I band (Figure 4c,d) and fitting the data with Gaussian functions (Figure 4e,f). The native Cyt c displayed a prominent band at ≈1653 cm^−1^, indicating a high content of α‐helix structure, which accounted for 66.8% of the total structure. However, the immobilized Cyt c within HZIF‐8 exhibited a noticeable decrease in the intensity of this band (40.5%), suggesting a degree of relaxation in the protein structure (Table S7, Supporting Information). These findings align with previously reported correlations between the activity of Cyt c and its α‐helix content.^[^ ^10^ ^]^

Solid‐state NMR spectroscopy (ssNMR) was further employed to investigate the intermolecular interactions between Cyt c and HZIF‐8. Initially, the 1D carbon‐13 cross‐polarization magic angle spinning NMR (^13^C CPMAS NMR) analysis of HZIF‐8@Cyt c revealed distinct peaks corresponding to the pristine HZIF‐8 structure (Figure S19, Supporting Information). Cyt c exhibited an NCO peak at 175 ppm, along with a less intense region below 90 ppm, encompassing the Hα as well as CH_3_/Cβ carbons (zoomed‐in region in Figure S19, Supporting Information). These peaks are absent in neat HZIF‐8. Subsequently, a 2D ^13^C‐^1^H heteronuclear correlation (HETCOR) NMR experiment was performed to investigate the interaction between Cyt c and HZIF‐8. In Figure 4g, cross‐correlation peaks were observed between the ^1^H NMR signals of Cyt c (CH_3_/Hβ ≈1.1 ppm) and the ^13^C species of the HZIF‐8 (160–120 ppm). The relative intensity of 1D slices extracted from the 2D spectra at the aromatic H position of 6.32 ppm corresponds to the cross‐correlation between Cyt c protons and HZIF‐8 carbon, as well as the self‐correlation signal from the HZIF‐8 aromatic protons, respectively. These findings provide evidence of intermolecular interaction between Cyt c and HZIF‐8_[Cyt c]_. These data compellingly demonstrate physical imprinting stabilizes active conformations through nanostructural effects that subtly remodel heme ligation, alter folding dynamics, and establish immobilizing intermolecular contacts. The exquisite architectural tailoring maximizes such bioactivity‐enhancing host–guest synergy at the molecular scale.

While our study offers compelling evidence of interactions between enzyme and ZIF framework, these interactions alone may not fully account for the observed enhancement in catalytic activity. Several key factors contribute significantly to this enhancement. The physical imprinting approach stabilizes active conformations by nanoscale architectural tuning within the mesopores, moving beyond simple surface immobilization. This subtly remodels influences active sites through constrained folding dynamics and heme ligation influenced by the tailored pore environment. Conformational changes are amplified inside the meticulously crafted mesopores, altering protein configurations and contacts with cofactors in a manner conducive to catalytic turnover. Furthermore, the intimate contacts established through imprinting optimize interactions at the molecular scale, finely modulating the enzyme microenvironment. By comparison, physical adsorption alone may not provide the exquisite pore‐level structuring afforded by our method. Only through judicious architectural manipulation within the constraints of the mesopores can such synergism be realized, surpassing the effects of basic tethering or encapsulation. Considering these various organizational effects in concert helps provide a more nuanced understanding of how the interplay between enzyme and scaffold is harnessed to enhance catalytic output. The meticulous tuning of conformation, dynamics, and contacts at the nanoscale likely underpins the pronounced activity gains observed.

## 3 Conclusion

Through physical imprinting, we have demonstrated the exquisite tuning of MOF architectures to achieve selective size‐ and shape‐complementary encapsulation of various enzymes. HZIF matrices imprinted by Cyt c, HRP, and lipase exhibited preferential uptake of their respective templating proteins. Rigorous characterization confirmed immobilization within customized mesopores, avoiding surface adsorption weaknesses. Strikingly, all imprinted biocatalysts surpassed free and de novo immobilized versions in activity, most profoundly Cyt c augmented over 1600%. Mechanistic insights proved physical imprinting subtly reshapes protein structure through nanoconfinement, stabilizing catalytically productive conformations without destabilization. Additionally, remarkable thermal, chemical, and operational resilience emerged from the optimized host‐guest synergy. Lipase impressively boosted catalytic efficiency 14‐fold. This comprehensive study illuminates physical imprinting as a generalizable and powerful methodology for engineering robust, hyperactive biocatalytic systems. By sensitively tailoring the immobilization interface through bottom‐up synthesis, full enzymatic potential can be unlocked and systematically correlated with architectural variables. Looking ahead, further optimizing imprinted MOFs holds great promise for advancing sustainable biomanufacturing and biotechnology. Achieving complete structure‐function resolution could revolutionize biocatalyst design. Our approach offers new avenues for developing next‐generation enzyme‐MOF synergies with transformational capabilities.

## 4 Experimental Section

### Materials and Reagents

Zinc nitrate hexahydrate, 2,2′‐azino‐bis (3‐ethylbenzothiazoline‐6‐sulfonic acid) (ABTS), hydrogen peroxide (H_2_O_2_), and 2‐methylimidazole were purchased from J&K Scientific Ltd. (Beijing, China). Cytochrome c from cow heart, horseradish peroxidase (lyophilized, HRP), and lipase were bought from Sigma–Aldrich (St. Louis, MO, USA), bovine serum albumin (BSA) were bought from Solarbio Life Sciences (Beijing, China). All other chemicals were obtained from Sinopharm Chemical Reagent Co., Ltd (Beijing, China).

### Instrumentation

Scanning electron microscopy images were taken with a JEOL JSM‐6700F field emission scanning electron microscope (Hitachi High‐Technologies, Tokyo, Japan). Powder X‐ray diffraction patterns were collected using a Bruker D8 Advance (Bruker Corporation, Germany). Nitrogen adsorption/desorption isotherms and pore size distributions were measured at 77 K using a V‐Sorb2800P surface area and porosimetry analyzer (Gold APP Instruments Corporation, Beijing, China). High‐resolution transmission electron microscopy (TEM) images and TEM energy‐dispersive X‐ray spectroscopy (EDS) mapping were acquired using a JEOL 2100F transmission electron microscope (Hitachi, Ltd., Japan). The thermal gravimetric analysis was carried out using a Thermogravimetric Analyzer (TGA) (Perkin Elmer, Pyris Diamond S(II), USA) at a heating rate of 10 °C min^−1^ in a temperature range of 25–800 °C under a nitrogen atmosphere (nitrogen flow rate 20 mL min^−1^). The UV–vis spectrum was carried out using a UV‐3600 UV–vis spectrophometer (Shimadzu, Japan). The low‐temperature electron paramagnetic resonance (EPR) experiments were conducted using a Bruker E500 EPR spectrometer equipped with a liquid nitrogen cryostat capable of reaching temperatures down to 90 K. The 1D and 2D solid‐state ^13^C NMR experiments were carried out on a Bruker AVANCE III 600 spectrometer with a 14.1 Tesla superconducting magnet operating at frequencies of 600 and 150 MHz for the ^13^C nuclei. The sample was packed into 4 mm zirconia rotors fitted with a Kel‐F cap and spun to 10 and 12 kHz at the magic angle (MAS) with recycle delays of 3 s. The 2D ^13^C‐^1^H heteronuclear correlation (HETCOR) spectrum was acquired at a contact time of 1 ms with 500 transients in the direct (F2) dimension and 70 increments of 21 µs each in the indirect (F1) dimension. The ^13^C chemical shifts were referenced to TMS.

### Synthesis of Enzyme@ZIF‐8 Composite

Zinc nitrate hexahydrate (0.57 g, 1.9 mmol) and 2‐methylimidazole (1.0 g, 12.2 mmol) were separately dissolved in 10 mL methanol. The solution was then subjected to ultrasonic treatment for 10 min to ensure complete dissolution and degassing. Different enzymes (Cyt c, HRP, lipase, BSA) were dissolved in 10 mL of deionized water as templates and added dropwise to the mixture of the first two solutions. The resulting mixture was stirred at room temperature for 24 h. Afterward, the obtained enzyme@ZIF‐8 composites were collected by centrifugation and then dried under vacuum at room temperature for 24 h.

### Synthesis of HZIF‐8_[enzyme]_

HZIF‐8_[enzyme]_ was obtained by calcinating the above synthesized enzyme@ZIF‐8 composites in a tubular furnace at 350 °C for 4 h under air atmosphere, the heating rate was 10 °C min^−1^ and the amount of calcination per batch was 80 mg.

### Immobilization of Enzymes in HZIF‐8_[enzyme]_

A 0.5 mL (1 mg mL^−1^) enzyme aqueous solution in 0.01 m phosphate buffer saline (PBS, pH 7.0) was added to 0.5 mL (4 mg mL^−1^) of HZIF‐8_[enzyme]_ suspension, and incubated for 5 h at 0 °C under stirring. After immobilization, the immobilized enzyme was separated by centrifugation and washed three times with PBS buffer (0.01 m, pH 7.0) to remove the surface‐adsorbed enzymes. The enzyme immobilization capacity was determined by calculating the residual protein solution in the supernatant.

### Determination of Enzyme Activity—Enzyme Activity of Cyt c

With H_2_O_2_ and ABTS of different concentrations as substrates, the peroxidase catalytic properties of Cyt c were utilized to oxidize ABTS to ABTS▪^+^, thus producing characteristic absorption at 415 nm, and the standard enzyme activity curve of Cyt c was drawn accordingly. 140 µL of H_2_O_2_ (5 mm) and 30 µL of ABTS substrate solution (5 mm) were added to 30 µL of Cyt c (1 µm, 12.3 µg) solution at room temperature and incubated for 2 min. The absorbance at 415 nm was measured to determine the enzyme activity. The enzyme activity of Cyt c was calculated using a standard curve. The determination method for immobilized Cyt c was the same, with the addition of an equal amount of immobilized enzyme.

### Enzyme Activity of HRP

In the 96‐well plate, 140 µL guaiacol solution (4 mm), 30 µL H_2_O_2_ solution (1.5 mm), and 30 µL HRP enzyme solution (0.002 mg mL^−1^, 0.06 µg) were mixed and incubated for 2 min. All the reactants listed above were dissolved in 0.01 m PBS. After 2 min of reaction, the absorbance of the reactants at 436 nm was measured. The enzyme activity was determined using HRP's standard curve of enzyme activity. The procedure for determining immobilized HRP was the same as this, with the addition of the same amount of immobilized enzyme.

### Enzyme Activity of Lipase

PBS (1.5 mL, pH 8) and 0.1 mL lipase solution (1 mg mL^−1^, 0.1 mg) were mixed into 0.1 mL isopropyl alcohol solution containing 0.33 mg mL^−1^ 4‐nitrophenyl laurate. After 6 min of reaction with the above reactants, a 2 mL ethanol cold water bath was added to stop the reaction. The product's absorbance at 410 nm was measured. The standard curve of lipase enzyme activity was used to calculate enzyme activity. The procedure for determining immobilized lipase was the same as this, with the exception of providing the same amount of immobilized enzyme.

### Kinetics Study of Cyt c

The study of cascade kinetics of Cyt c immobilized within HZIF‐8 compared to free Cyt c in solution was carried out in a 200 µL clear 96‐well plate using H_2_O_2_ and ABTS as substrates. The H_2_O_2_ solution with concentrations ranging from 5 to 500 mmol L^−1^ was added to the enzyme system containing 5 mmol L^−1^ ABTS. The absorbance at 415 nm of the product was recorded.

### Kinetics Study of HRP

The study of cascade kinetics of HRP immobilized within HZIF‐8 compared to free HRP in solution was carried out in a 200 µL clear 96‐well plate using guaiacol and H_2_O_2_ as substrates. The guaiacol solution with concentrations ranging from 0.2 to 10 mmol L^−1^ was added to the enzyme system containing 5 mmol L^−1^ H_2_O_2_. The absorbance at 436 nm of the product was recorded.

### Kinetics Study of Lipase

The study of cascade kinetics of lipase immobilized within HZIF‐8 compared to free lipase in solution was carried out using 4‐nitrophenyl laurate as substrates. The 4‐nitrophenyl laurate solution with concentrations ranging from 0.2 to 100 mmol L^−1^ was added to the enzyme system. The absorbance at 410 nm of the product was recorded.

### Operational Stability Study

The store solutions of free enzymes (Cyt c, HRP, and lipase) and immobilized enzymes (enzyme@HZIF‐8 dispersing in 1 mL phosphate buffer saline at pH 7.0) were first prepared.

To investigate the effects of temperature on the residual enzymatic activity, the enzyme solutions were left at different temperatures of 45, 65, and 95 °C for 2 h. After the solution had cooled to room temperature, the substrate solution was added into the above solution. The corresponding enzyme‐catalyzed reaction was performed and the OD values at 415, 436, and 410 nm were detected to calculate the residual enzyme activity.

To study the effects of pH values on residual enzymatic activity, the enzyme solutions with a concentration of 2 mg mL^−1^ were subjected to varying pH conditions and allowed to equilibrate at room temperature for a duration of 3 h. Subsequently, the pH of the enzyme solutions was standardized to neutral using 10 mM phosphate‐buffered saline (pH 7.2–7.4). Following this adjustment, the substrate solution was introduced to the aforementioned solutions. The corresponding enzyme‐catalyzed reaction was performed, and the OD values at 415, 436, and 410 nm were detected to calculate the residual enzyme activity.

To evaluate the thermal and chemical stabilities of immobilized enzymes under extreme conditions, the enzyme solutions were incubated with methanol, acetone, and trypsin for 8 h. Then, the substrate solution was added into the above solution. The corresponding enzyme‐catalyzed reaction was performed and the OD values at 415, 436, and 410 nm were detected to calculate the residual enzyme activity.

## Conflict of Interest

The authors declare no conflict of interest.

## Supporting information

Supporting Information

## Data Availability

The data that support the findings of this study are available in the supplementary material of this article.
