# Supplementary material for: Boosted Enzyme Activity via Encapsulation within Metal–Organic Frameworks with Pores Matching Enzyme Size and Shape
Source: Adv Sci (Weinh). 2024 Apr 4;11(21):2309243. doi: 10.1002/advs.202309243 (PMC11151065; doi:10.1002/advs.202309243)
Supplement: Supplementary file 1 — Supporting Information [file ADVS-11-2309243-s001.pdf]

ADVANCED SCIENCE
Open Access

Supporting Information

for *Adv. Sci.*, DOI 10.1002/advs.202309243

Boosted Enzyme Activity via Encapsulation within Metal–Organic Frameworks with Pores Matching Enzyme Size and Shape

Ying Liu, Ziman Chen, Zheng Wang and Yongqin Lv**

Supporting Information

Boosted Enzyme Activity through Encapsulation in a Metal-Organic Frameworks with Pores Matching Enzyme Size and Shape

Ying Liu,[1] Ziman Chen,[1] Zheng Wang, Yongqin Lv **

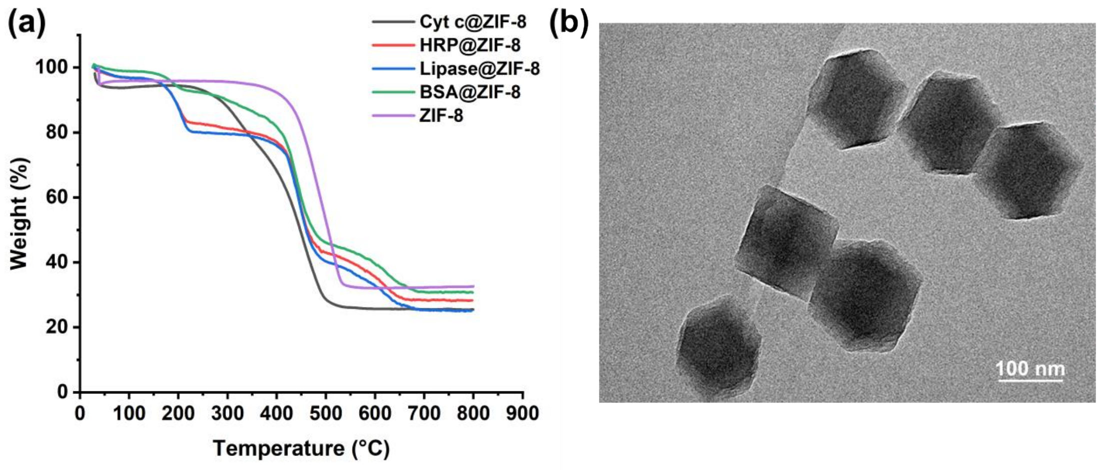

Figure S1. (a) Thermal gravimetric analysis (TGA) of Cyt C@ZIF-8, HRP@ZIF-8, lipase@ZIF-8, BSA@ZIF-8, and ZIF-8. (b) Transmission electron microscopy (TEM) image of conventional microporous ZIF-8 after calcination at 350°C for 4 h.

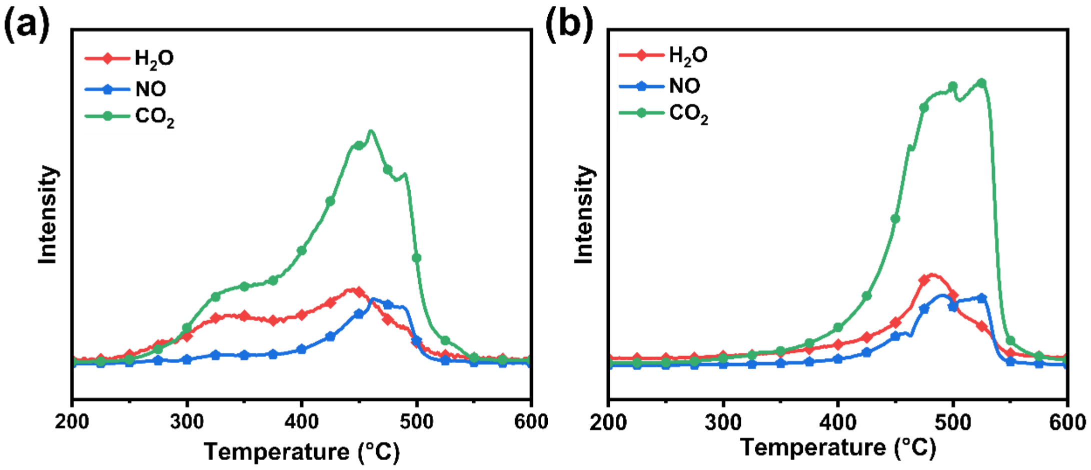

Figure S2. The online thermogravimetry-mass spectrometry curves of Cyt C-ZIF-8 composite (a) and conventional microporous ZIF-8 (b).

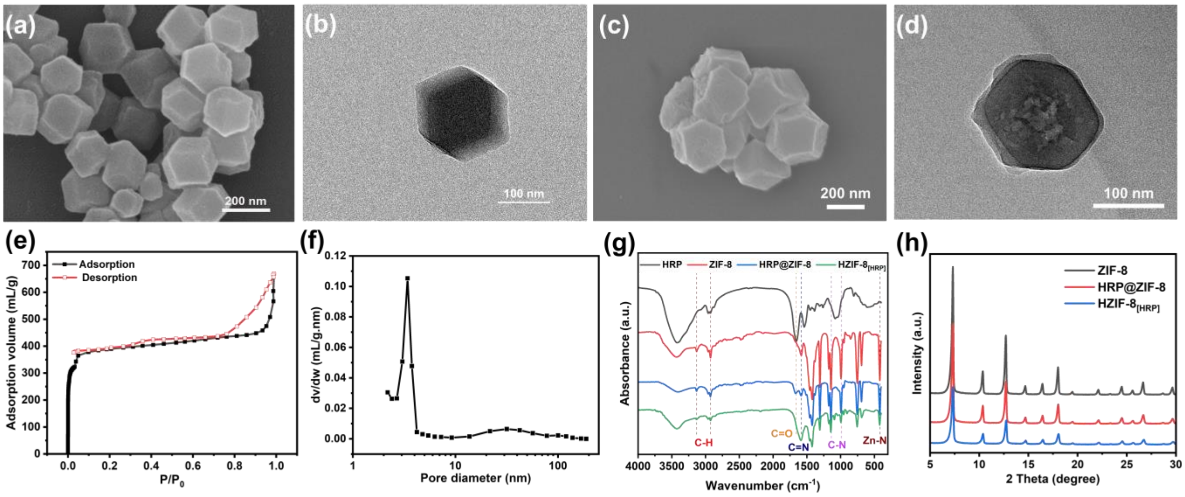

Figure S3. (a) SEM image of HRP@ZIF-8 composite; (b) TEM image of HRP@ZIF-8 composite; (c) SEM image of HZIF-8$_{[HRP]}$; (d) TEM image of HZIF-8$_{[HRP]}$; (e) N_2 adsorption/desorption isotherms of HZIF-8$_{[HRP]}$; (f) Pore size distribution of HZIF-8$_{[HRP]}$; (g) FTIR spectra of HRP, ZIF-8, HRP@ZIF-8 composite, and HZIF-8$_{[HRP]}$; (h) PXRD of ZIF-8, HRP@ZIF-8 composite, and HZIF-8$_{[HRP]}$.

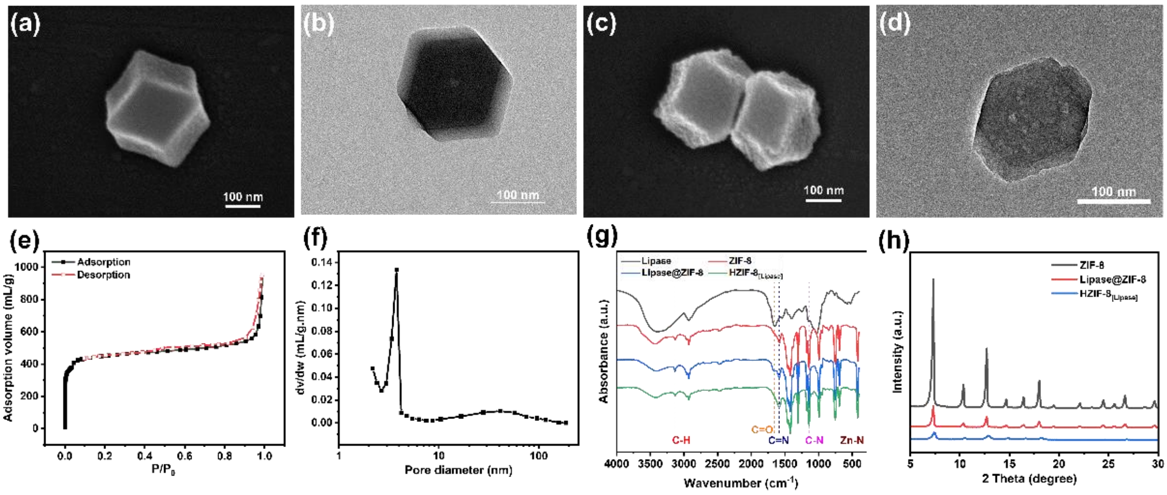

Figure S4. (a) SEM image of lipase@ZIF-8 composite; (b) TEM image of lipase@ZIF-8 composite; (c) SEM image of HZIF-$8_{[Lipase]}$; (d) TEM image of HZIF-$8_{[Lipase]}$; (e) N_2 adsorption/desorption isotherms of HZIF-$8_{[Lipase]}$; (f) Pore size distribution of HZIF-$8_{[Lipase]}$; (g) FTIR spectra of lipase, ZIF-8, lipase@ZIF-8 composite, and HZIF-$8_{[Lipase]}$; (h) PXRD of ZIF-8, lipase@ZIF-8 composite, and HZIF-$8_{[Lipase]}$.

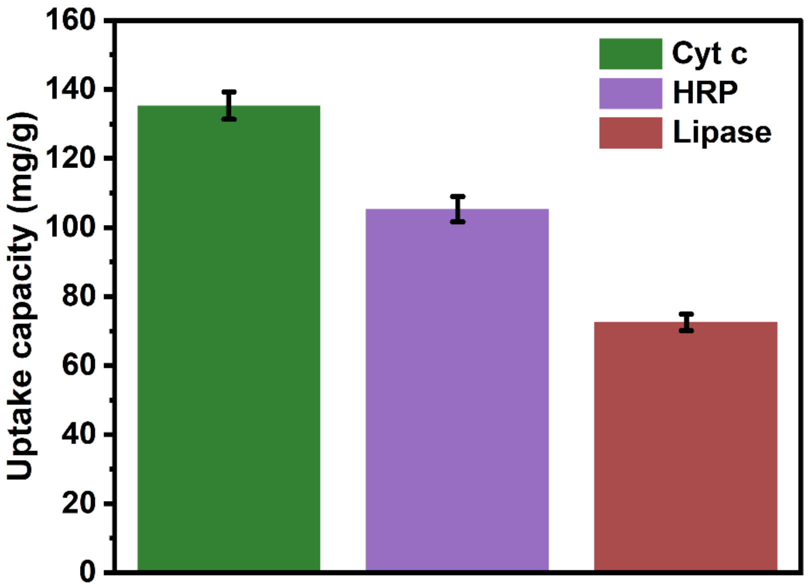

Figure S5. Uptake capacity of HZIF-$8_{[BSA]}$.

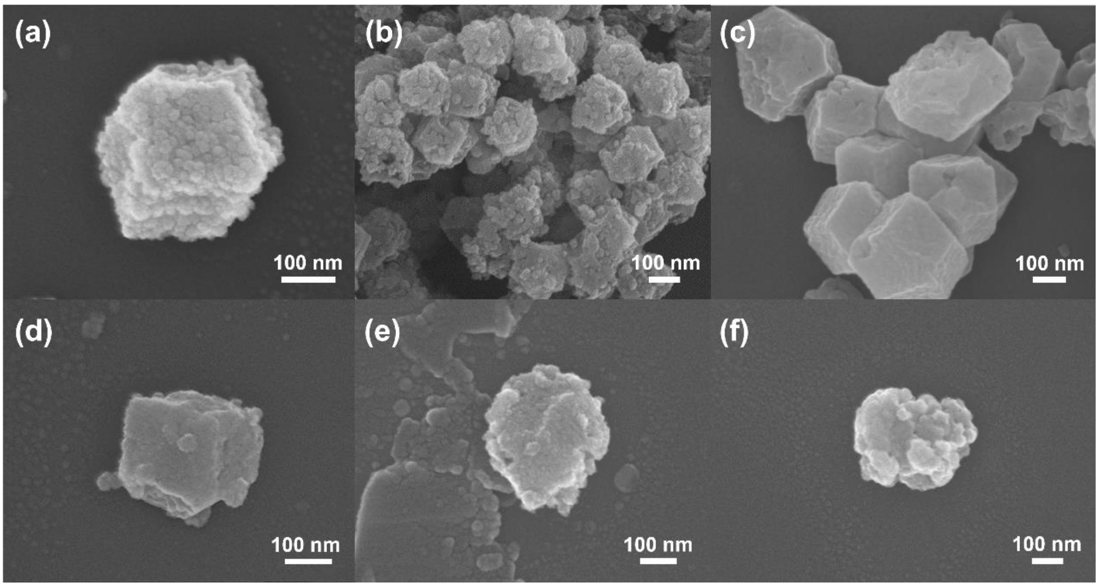

Figure S6. SEM image of Cyt c@HZIF-8$_{[Cyt\ c]}$ (a), HRP@HZIF-8$_{[HRP]}$ (b), lipase@HZIF-8$_{[Lipase]}$ (c), Cyt c@HZIF-8$_{[BSA]}$ (d), HRP@HZIF-8$_{[BSA]}$ (e), and lipase@HZIF-8$_{[BSA]}$ (f).

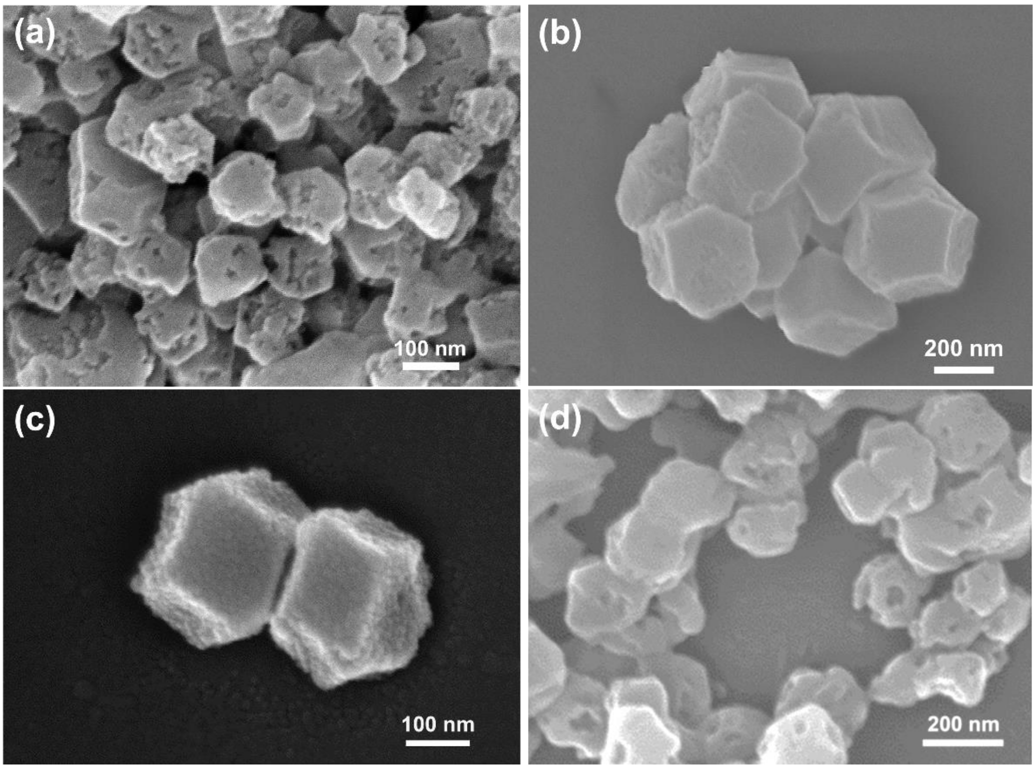

Figure S7. The enlarged SEM image of HZIF-8$_{[Cyt\ c]}$ (a), HZIF-8$_{[HRP]}$ (b), HZIF-8$_{[Lipase]}$ (c), HZIF-8$_{[BSA]}$ (d)

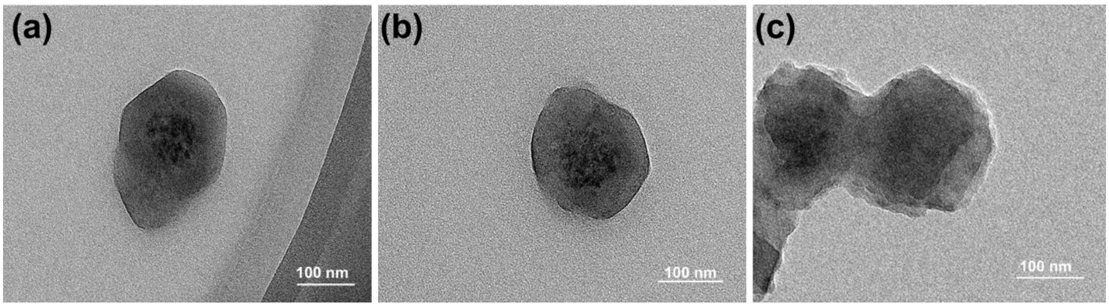

Figure S8. Transmission electron microscopy (TEM) images of Cyt c@HZIF-$8_{[BSA]}$ (a), HRP@HZIF-$8_{[BSA]}$ (b), and lipase@HZIF-$8_{[BSA]}$ (c).

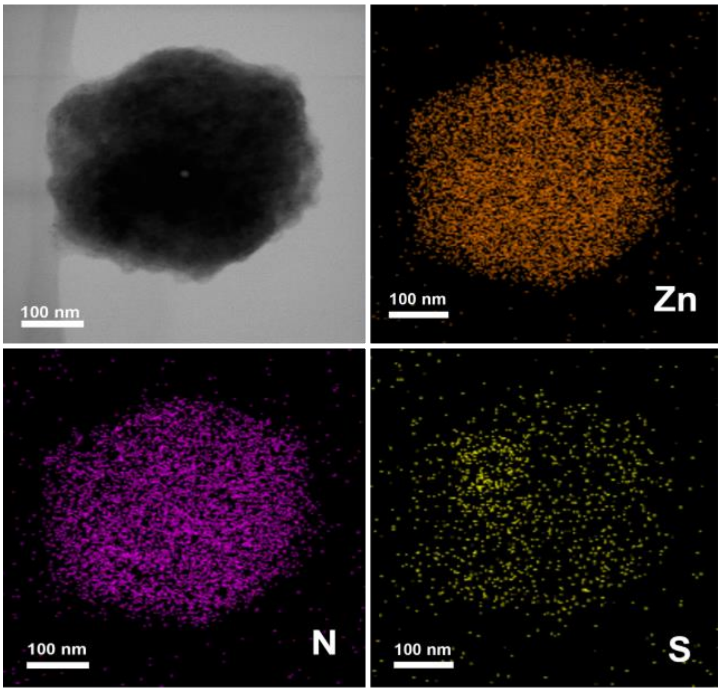

Figure S9. Energy dispersive spectroscopy (EDS) of Cyt c@HZIF-$8_{[Cyt\ c]}$ for elemental mapping in TEM.

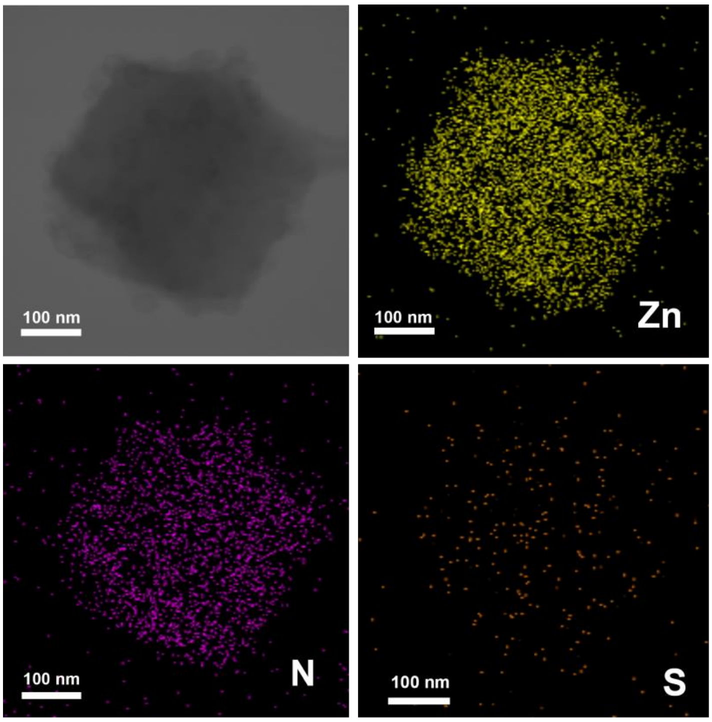

Figure S10. Energy dispersive spectroscopy (EDS) of HRP@HZIF-$8_{[HRP]}$ for elemental mapping in TEM.

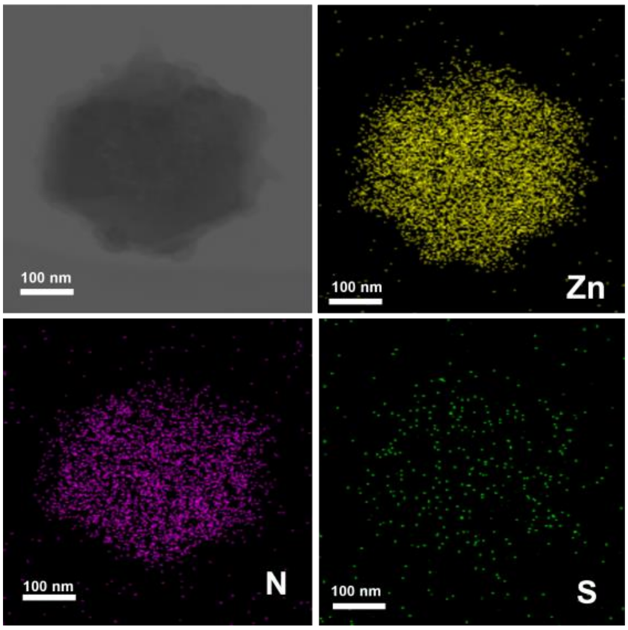

Figure S11. Energy dispersive spectroscopy (EDS) of lipase@HZIF-$8_{[Lipase]}$ for elemental mapping in TEM.

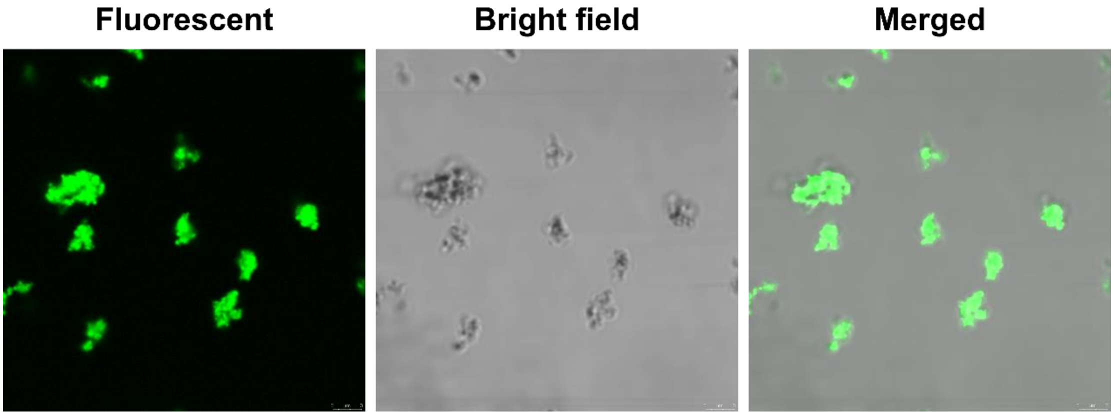

Figure S12. CLSM images of Cyt c@HZIF-8$_{[Cyt\ c]}$ (Cyt c was labeled with FITC).

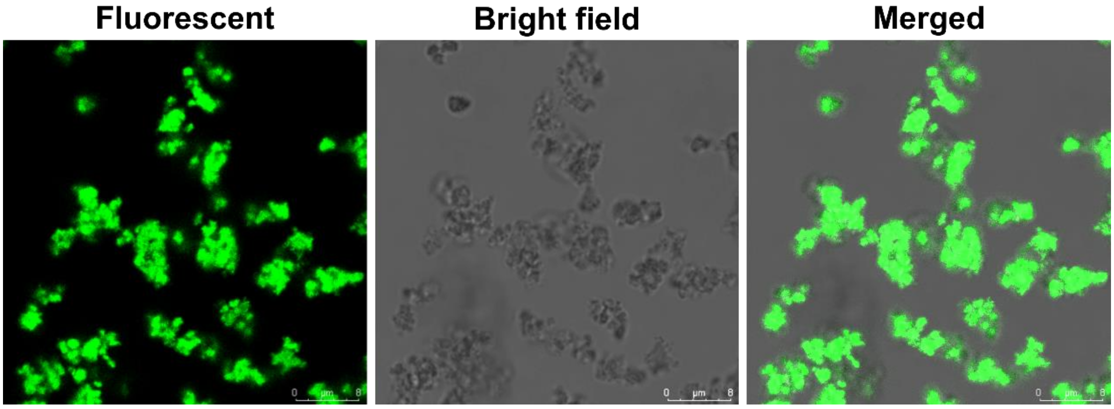

Figure S13. CLSM images of HRP@HZIF-8$_{[HRP]}$ (HRP was labeled with FITC).

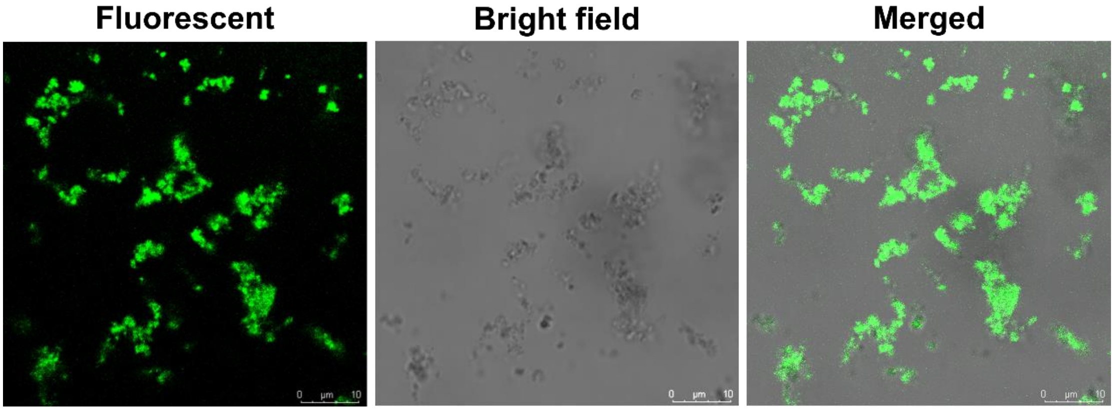

Figure S14. CLSM images of Lipase@HZIF-8$_{[Lipase]}$ (Lipase was labeled with FITC).

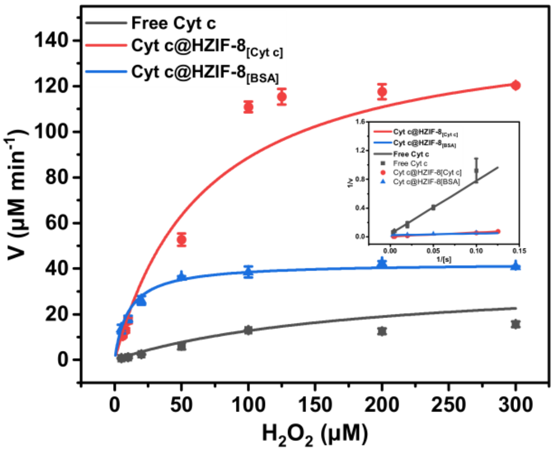

Figure S15. Kinetics study of Cyt c@HZIF-8$_{[Cyt\ c]}$, Cyt c@HZIF-8$_{[BSA]}$, and free Cyt C (a) and their corresponding Lineweaver-Burk plots (b).

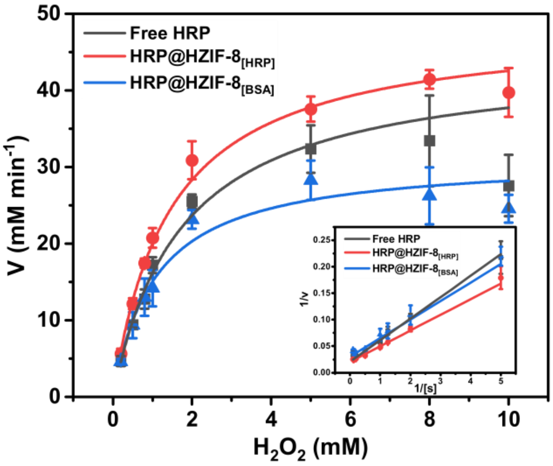

Figure S16. Kinetics study of HRP@HZIF-8$_{[HRP]}$, HRP@HZIF-8$_{[BSA]}$, and free HRP (a), and their corresponding Lineweaver-Burk plots (b).

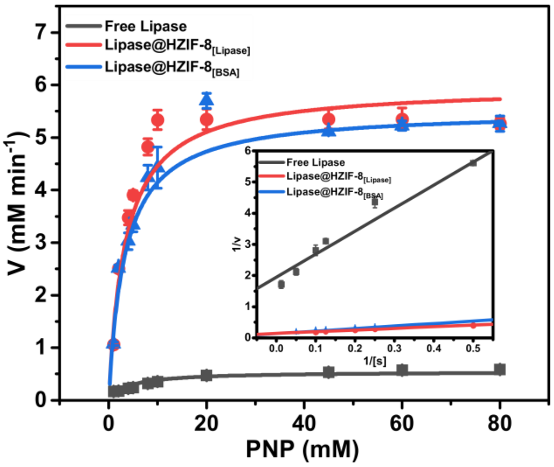

Figure S17. Kinetics study of lipase@HZIF-8$_{[Lipase]}$, lipase@HZIF-8$_{[BSA]}$, and free lipase (a), and their corresponding Lineweaver-Burk plots (b).

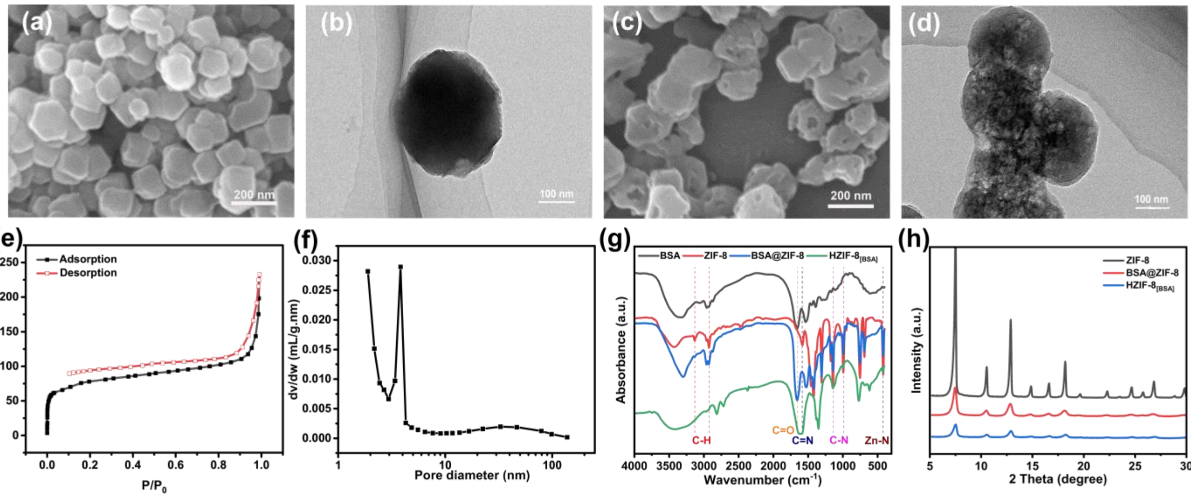

Figure S18. SEM images of BSA@ZIF-8 composite (a) and HZIF-8$_{[BSA]}$ (c). TEM images of BSA@ZIF-8 composite (b) and HZIF-8$_{[BSA]}$ (d). N_2 adsorption/desorption isotherms of HZIF-8$_{[BSA]}$ (e). Pore size distribution of HZIF-8$_{[BSA]}$ (f). FTIR spectra of BSA, ZIF-8, BSA@ZIF-8, and HZIF-8$_{[BSA]}$ (g). PXRD patterns of ZIF-8, BSA-ZIF-8 and HZIF-8$_{[BSA]}$ (h).

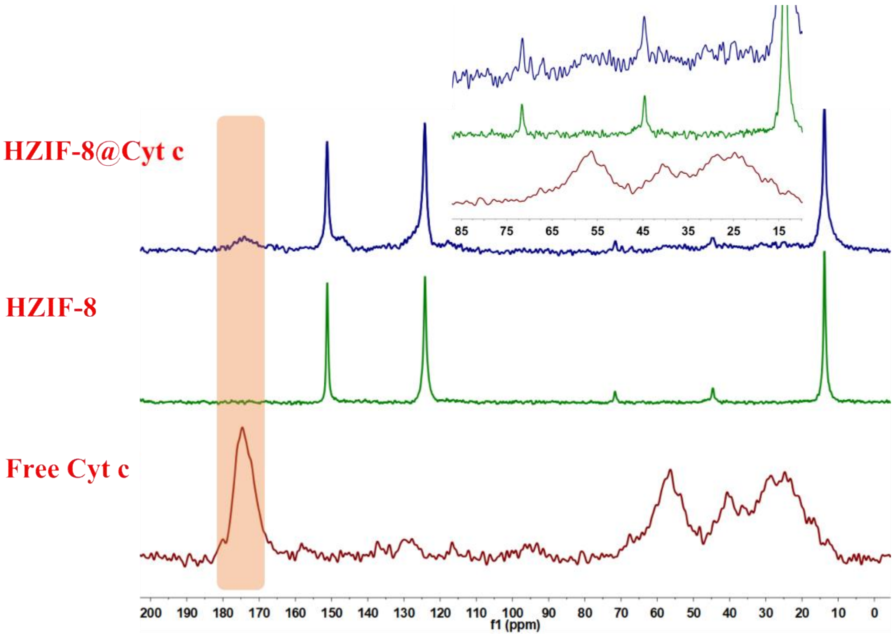

Figure S19. Solid-state ^{13}C CP-MAS NMR spectra of free Cyt c, HZIF-8 and Cyt c@HZIF-8$_{[Cyt\ c]}$.

Table S1. Porous properties of HZIF-8 prepared using the physical imprinting strategy with different enzymes as templates.

MOF	Template	Template molecular weight (kDa)	Template dimension (nm)	Average pore width [nm]		Pore volume [mL g^{-1}]		Surface area [m^2 g^{-1}]
				0-2 nm[a]	2-50 nm[b]	0-2 nm[a]	2-50 nm[b]	
HZIF-8$_{[Cyt\ c]}$	Cyt c	12.3	3.2×2.7×3.8	0.76	7.90	0.28	0.48	674.6
HZIF-8$_{[HRP]}$	HRP	40.0	4×4.4×6.8	0.71	11.89	0.58	0.51	1264.3
HZIF-8$_{[Lipase]}$	Lipase	48.0	6.2×5.5×14.4	0.78	15.04	0.67	0.88	1494.9
HZIF-8$_{[BSA]}$	BSA	66.4	4×4×14	0.70	20.15	0.14	0.24	277.3

[a] The average micropores width and pore volume of MOFs were calculated according to the SF method; [b] The average mesopores width and pore volume of MOFs were calculated according to the BJH method.

Table S2. Pore volume distribution across different pore sizes in HZIF-8$_{[Cyt\ c]}$.

MOF	Pore volume proportion [%]					
	2-3.8 nm	3.8-7.6 nm	7.6-11.4 nm	11.4-15.2 nm	15.2-19 nm	>19 nm
HZIF-8$_{[Cyt\ c]}$	21.8	14.9	8.4	5.8	11.1	38.0

Table S3. Pore volume distribution across different pore sizes in HZIF-8$_{[BSA]}$.

MOF	Pore volume proportion [%]					
	2-6.8 nm	6.8-13.6 nm	13.6-20.4 nm	20.4-27.2 nm	27.2-34 nm	>34 nm
HZIF-8$_{[HRP]}$	19.3	1.2	3.3	13.3	10.4	52.5

Table S4. Pore volume distribution across different pore sizes in HZIF-8$_{[Lipase]}$.

MOF	Pore volume proportion [%]					
	2-14.4 nm	14.4-28.8 nm	28.8-43.2 nm	43.2-57.6 nm	57.6-72 nm	>72 nm
HZIF-8$_{[Lipase]}$	16.8	18.7	18.8	15.4	8.8	21.5

Table S5. Pore volume distribution across different pore sizes in HZIF-8$_{[BSA]}$.

MOF	Pore volume proportion [%]					
	2-14	14-28 nm	28-42 nm	52-56 nm	56-70 nm	>70 nm
HZIF-8$_{[BSA]}$	24.2	8.6	5.6	22.3	4.8	34.5

Table S6. Kinetic parameters of cascade reaction catalyzed by free and immobilized enzymes.

Catalyst	K_m (mM)	k_{cat} (s^{-1})	k_{cat}/K_m (mM^{-1} s^{-1})	Relative activity (%)	Refs
Free Cyt c	0.6	0.71	1.18	100	[1]
Cyt c@ZIF-8	0.54	0.047	0.087	9.5	[1]
Cyt c-A@ZIF-8	1.19	0.56	0.47	73.5	[1]
Free Cyt c	19.49	0.78	0.04	100	[2]
Cyt c-LDAO	1.86	0.11	0.06	140	[2]
Cyt c-MicroCC3	1.21	0.05	0.04	100	[2]
Cyt c@MesoCC3-LDAO	0.49	0.09	0.19	480	[2]
Free Cyt c	123	0.984	0.008	100	[3]
Cyt c@NU-1000	20	0.2	0.010	560	[3]
Free Cyt c	58	---	---	100	[4]
Cyt c@$[EP]_{40\%}$-TD-COF	29.2	---	---	600	[4]
Free Cyt C_{US} [a]	---	---	---	162	[5]
Cyt C_{US}-ZIF-8 [a]	29	412.82×10^{6}	14.24×10^{6}	913	[5]
Free Cyt c	15	---	---	100	[6]
Cyt c@ZIF-8	2	---	---	1130	[6]
Free Cyt c	48.3	1.1	0.023	100	[7]
Cyt c@PCN-333(Al)	14.9	0.54	0.036	1450	[7]
Free Cyt c	183.1×10^{-3}	0.4	2.3	100	This work
Cyt c@HZIF-$8_{[Cytc]}$	84.4×10^{-3}	2.6	30.5	1674	This work
Cyt c@HZIF-$8_{[BSA]}$	9.8×10^{-3}	0.7	72.2	1206	This work

Free HRP	0.07	484.48	6921.1	100	[1]
HRP@ZIF-8	---	---	---	4.3	[1]
HRP-A@ZIF-8	0.46	9.28	20.2	82.9	[1]
Free HRP	---	---	---	100	[6]
HRP@ZIF-8	---	---	---	28	[6]
Free HRP	1.8	753.3	418.3	100	[7]
HRP@PCN-333(Al)	0.84	256.7	305.5	100.6	[7]
Free HRP	0.038	---	---	100	[8]
HRP@NMOF	0.045	---	---	90	[8]
Free HRP_{US} [a]	1.00	107.9×10^6	108.07×10^6	123	[5]
HRP_{US}-ZIF-8 [a]	0.20	8.97×10^6	44.86×10^6	214	[5]
Free HRP	1.96	107.1	54.8	100	This work
HRP@HZIF-$8_{[HRP]}$	1.44	108.3	75.3	94.5	This work
HRP@HZIF-$8_{[BSA]}$	1.15	73.2	63.7	112.8	This work
Free lipase	---	---	---	100	[6]
Lipase@ZIF-8	---	---	---	88	[6]
Free Lipase	6.78×10^{-4}	---	---	100	[9]
Lipase-SDS@ZIF-8	5.23×10^{-4}	---	---	253	[9]
Free lipase	---	---	---	100	[10]
Lipase@Fe-BTC	---	---	---	97	[10]
Free lipase	11.22	---	---	100	[11]
Lipase@ZnGlu-MNP	2.16	---	---	82	[11]
Free lipase	3.80	7.0	1.8	100	This work
Lipase@HZIF-$8_{[Lipase]}$	3.77	95.3	25.3	191	This work

Lipase@HZIF-8[BSA]	5.95	99.9	16.8	140	This work

[a] The enzymes were first treated with ultrasound, and then encapsulated in ZIF-8 via a co-precipitation approach.

Table S7. Fractions of secondary structures for free Cyt c, HZIF-8@Cyt c.

	Assign.	v[a] (cm^{-1})	v[b] (cm^{-1})	Areas (%)
Cyt c	Intermolecular β-sheets	1614	1619	4.7
	β-sheets	1633	1628	2.9
	random	1639	1636	4.1
	α-helix	1656	1653	66.8
	β-turns	1664<br>1678<br>1683	1669<br>1683	21.5
HZIF-8@Cyt c	Intermolecular β-sheets	1614	1613	1.7
	β-sheets	1633	1627	10.1
	random	1639	1645	32.1
	α-helix	1656	1664	40.5
		1664	1679	11.0
	β-turns	1678	1688	3.7
		1683	1694	0.9

[a] The peak position of the secondary structure was determined by the second derivative of the amide I band; [b] The position of multi-component peak was obtained by curve fitting.

Reference

[1] G. Chen, X. Kou, S. Huang, L. Tong, Y. Shen, W. Zhu, F. Zhu, G. Ouyang, *Angew. Chem. Int. Ed.* **2020**, 59, 2867.

[2] M. Hua, S. Wang, Y. Gong, J. Wei, Z. Yang, J.-K. Sun, *Angew. Chem. Int. Ed.* **2021**, 60, 12490.

[3] Y. Chen, F. Jiménez-Ángeles, B. Qiao, M. D. Krzyaniak, F. Sha, S. Kato, X. Gong, C. T. Buru, Z. Chen, X. Zhang, N. C. Gianneschi, M. R. Wasielewski, M. Olvera de la Cruz, O. K. Farha, *J. Am. Chem. Soc.* **2020**, 142, 18576.

[4] C. Xing, P. Mei, Z. Mu, B. Li, X. Feng, Y. Zhang, B. Wang, *Angew. Chem. Int. Ed.* **2022**, 61, e202201378.

[5] J. Liang, M. Y. Bin Zulkifli, J. Yong, Z. Du, Z. Ao, A. Rawal, J. A. Scott, J. R. Harmer, J. Wang, K. Liang, *J. Am. Chem. Soc.* **2022**, 144, 17865.

[6] F. Lyu, Y. Zhang, R. N. Zare, J. Ge, Z. Liu, *Nano Lett.* **2014**, 14, 5761.

[7] D. Feng, T.-F. Liu, J. Su, M. Bosch, Z. Wei, W. Wan, D. Yuan, Y.-P. Chen, X. Wang, K. Wang, X. Lian, Z.-Y. Gu, J. Park, X. Zou, H.-C. Zhou, *Nat. Commun.* **2015**, 6, 5979.

[8] W.-H. Chen, M. Vázquez-González, A. Zoabi, R. Abu-Reziq, I. Willner, *Nature Catalysis* **2018**, 1, 689.

[9] L. B. Vaidya, S. S. Nadar, V. K. Rathod, *Int. J. Biol. Macromol.* **2020**, 146, 678.

[10] a) V. Gascón, C. Carucci, M. B. Jiménez, R. M. Blanco, M. Sánchez-Sánchez, E. Magner, *ChemCatChem* **2017**, 9, 1182; b) V. Gascón, M. B. Jiménez, R. M. Blanco, M. Sanchez-Sanchez, *Catal. Today* **2018**, 304, 119.

[11] G. Xia, S.-L. Cao, P. Xu, X. Li, J. Zhou, M. Zong, *ChemCatChem* **2017**, 9.
